# Off to a good start after a cancer diagnosis: implementation of a time out consultation in primary care before cancer treatment decision

**DOI:** 10.1007/s11764-019-00814-5

**Published:** 2019-11-16

**Authors:** Eveline A. Noteboom, Niek J. de Wit, Ingrid J. E. M. van Asseldonk, Monique C. A. M. Janssen, Wai Yee Lam-Wong, Rob H. P. J. Linssen, Manon J. A. E. Pepels, Natascha A. W. P. Schrama, Mariëlle E. H. Trompper, L. Maaike Veldhuizen, Anne P. Wijtvliet, Ed G. F. Zeldenrust, Ans M. Hendrikx, Wil A. van de Boomen, Dorothé M. Elbersen, Esther M. G. Jacobs, Elsken van der Wall, Charles W. Helsper

**Affiliations:** 1 Julius Center for Health Sciences and Primary Care, University Medical Center Utrecht, Utrecht University, P.O. Box 85500, 3508 GA Utrecht, The Netherlands; 2 Quality of Life Group, Elkerliek Hospital, P.O. Box 98, 5700 AB Helmond, The Netherlands; 3 Department of Medical Oncology, University Medical Center Utrecht, Utrecht University, P.O. Box 85500, Utrecht, 3508 GA The Netherlands

**Keywords:** Decision-making, Medical oncology, Primary health care

## Abstract

**Purpose:**

Supportive care for cancer patients may benefit from improving treatment decisions and optimal use of the family physicians’ and specialists’ strengths. To improve shared decision-making (SDM) and facilitate continuity of primary care during treatment, a cancer care path including a “time out consultation” (TOC) in primary care before treatment decision, was implemented. This study assesses the uptake of a TOC and the added value for SDM.

**Methods:**

For patients with metastatic lung or gastro-intestinal cancer, a TOC was introduced in their care path in a southern region of The Netherlands, from April until October 2016. Uptake of a TOC was measured to reflect on facilitation of continuity of primary care. The added value for SDM and overall experiences were evaluated with questionnaires and semi-structured interviews among patients, family physicians, and specialists.

**Results:**

Of the 40 patients who were offered a TOC, 31 (78%) had a TOC. Almost all patients, family physicians, and specialists expressed that they experienced added value for SDM. This includes a stimulating effect on reflection on choice (expressed by 83% of patients) and improved preparation for treatment decision (75% of patients). Overall added value of a TOC for SDM, only evaluated among family physicians and specialists, was experienced by 71% and 86% of these physicians, respectively.

**Conclusion and Implications for Cancer Survivors:**

The first experiences with a TOC in primary care before cancer treatment decision suggest that it may help to keep the GP “in the loop” after a cancer diagnosis and that it may contribute to the SDM process, according to patients, family physicians, and specialists.

## Introduction

The rapid developments in cancer treatment have facilitated opportunities for personalized cancer care [1]. Consequently, the optimal balance between the benefits and harms of treatment is increasingly linked to individual preference. Unfortunately, the current “rollercoaster” cancer care pathway after diagnosis does not facilitate tailored treatment decisions, personalized to patient’s individual preferences [2]. Additionally, the consequences of treatment are frequently not fully understood by patients [3].

To enable personalized decision-making, facilitating shared decision-making (SDM) is key [4]. SDM encompasses several steps: (step 1) creation of awareness of choice, (step 2) sharing of treatment options, (step 3) time and space for deliberation to explore personal priorities, and (step 4) making an informed shared decision [4].

Involvement of the family physician may improve the SDM process [5, 6]. Family physicians generally have a long-standing, personal relationship with their patients, including knowledge of comorbidities and personal circumstances and values. However, keeping the family physician “in the loop” after a cancer diagnosis is currently insufficiently facilitated. This hinders possibilities for the family physician to support the SDM process and to safeguard personalized survivorship and supportive care [2, 5–7].

To improve personalized decision-making and facilitate continuity of primary care, we developed a cancer care pathway including a “time out consultation” (TOC) with the family physician. This TOC is scheduled between the cancer diagnosis and the corresponding treatment decision in secondary care. It aims to support patients in making an optimal treatment decision.

We performed a pilot implementation of a TOC for patients with metastatic gastro-intestinal or lung cancer. This pilot study aimed to explore uptake and first experiences with a TOC concerning experienced added value for SDM according to patients, family physicians, and specialists.

## Methods

### Study design

The implementation of the TOC in usual care was evaluated using a non-comparative intervention design, with questionnaires and semi-structured interviews among patients, family physicians, and specialists, from April to October 2016, by the Quality of Life Group, a collaboration of regional family physicians and the Elkerliek Hospital in Helmond, The Netherlands. 

### Study population

All patients visiting the Elkerliek Hospital from April to October 2016 with a new diagnosis of metastatic gastro-intestinal or lung cancer or with changes in treatment perspective (e.g. progression from localized cancer) facing a new treatment decision were offered a TOC by their specialist.

### Intervention: time out consultation

If the patient agreed, the specialist or oncology nurse contacted the family physician’s office. There, the assistant contacted the patient to plan the TOC. Before the TOC, the specialist provided the family physician with relevant information about diagnosis, treatment options including pros and cons, and if possible expected prognosis. The TOC consisted of a 20-min consultation with the patient’s family physician.

The TOC aimed to improve continuity of primary care and to support the SDM process. Suggested topics in the TOC were as follows: (1) impact and consequences of the diagnosis, (2) personal preferences and priorities in the light of the expected prognosis and options, and (3) providing three key questions to be asked during the follow-up consultation with the specialist: (a) What are my options? (b) What are the benefits and harms of these options? and (c) How likely are these benefits and harms to occur in my situation? Incorporating these questions in a treatment decision consultation previously demonstrated to improve the SDM process [8]. The family physician provided the patient with a form including these three questions and room for remaining questions. After the TOC, the family physician informed the specialist in case of relevant information. The treatment decision generally occurred approximately 1 week after the TOC procedure started.

A short TOC instruction text, describing the aim and proposed topics of the TOC, was available for the family physicians on the hospital website. All family physicians were informed by a newsletter about the new TOC care pathway, the TOC instruction text, and the study procedures, prior to the start of the pilot.

### Outcomes and measurements

Uptake of the TOC was defined as the percentage of patients who were offered a TOC, which actually visited the family physician for a TOC. Experienced added value of a TOC for the SDM steps (e.g. the benefit which was experienced by the physician for reflection on choice and preparation for treatment decision-making) was assessed using self-constructed, non-validated questionnaires, and semi-structured interviews. The questionnaires were sent to all patients who were offered a TOC. After each TOC and treatment decision consultation in the hospital, questionnaires were sent to the corresponding family physician and specialist. One family physician or specialist could potentially fill in multiple questionnaires evaluating different TOCs*.* Semi-structured interviews were conducted with a random sample of family physicians and patients. These aimed to explore general experiences. Answers to the open-ended questionnaire questions and data from the interviews were considered of comparable value. In these data, TOC-related quotes referring to any of the steps of SDM were marked. These quotes were categorized to evaluate added value for each SDM step. Only the second SDM step “sharing of treatment options” was not taken into account since treatment options are shared in the hospital and this is not a topic of the TOC.

### Ethical approval

All procedures performed were in accordance with the 1964 Helsinki Declaration, its amendments and comparable ethical standards. As the implementation concerned an evaluation of new standard practice, the Medical Research Human Subject Acts does not apply.

### Informed consent

Informed consent was obtained from all study participants.

## Results

### Uptake of TOC

**Fig. 1:**
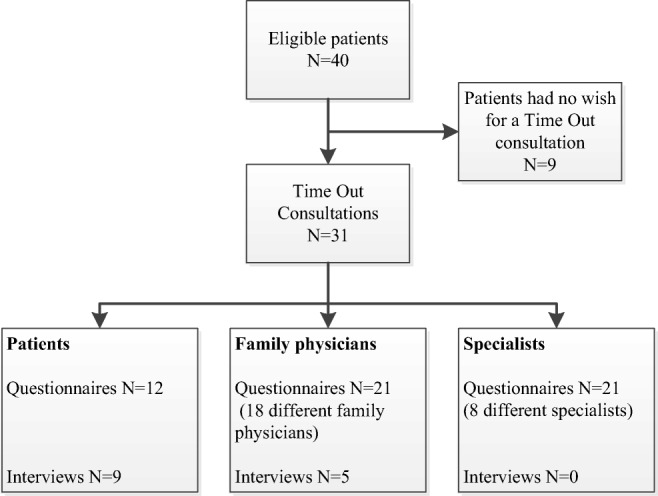
Number of eligible patients, time out consultations, received questionnaires from patients, family physicians and specialists, and number of interviews

Of 40 eligible patients, 31 (78%) visited their family physician for a TOC. Of these patients, 12 returned the questionnaires. We received 21 questionnaires from 18 family physicians evaluating 21 different TOCs and 21 questionnaires from 8 specialists evaluating 21 different TOCs. Semi-structured interviews were conducted with 9 patients and 5 family physicians (see Fig. 1).

**Fig. 2:**
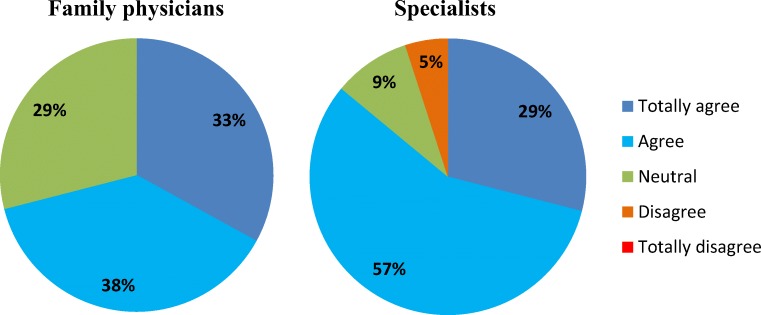
Answers of family physicians and specialists to the question if the time out consultation contributes to shared decision-making. Percentages are percentages of the number of consultations on which the family physicians (*N* = 21) and specialists (*N* = 21) reflect

Overall, added value of a TOC for SDM was experienced by family physicians in 15 out of 21 (71%) TOCs and by specialists in 18 out of 21 (86%) TOCs (Fig. 2).

### SDM step 1—Awareness of choice

Patients described that the TOC created the awareness that they make the final decision.

“We discussed the treatment options and it was communicated clearly that the choice is with me.” (Patient)

Family physicians indicated that awareness was created for the option not to treat and that a TOC can reduce the risk of choosing the therapy preferred by the specialist out of loyalty, instead of the patients’ own preference.

### SDM step 3—Deliberation

For the majority of the TOCs, patients (10/12) and family physicians (14/21) experienced that the “TOC had added value for reflection on treatment decision.” Additionally, in most TOCs, patients (9/12) and about half of the family physicians (11/21) experienced that “the patient is better prepared for the treatment decision consultation by the TOC.”

The qualitative data show that according to patients, preparation for treatment decision included (1) discussing patient’s wishes, (2) creating clarity on possible treatment options, (3) asking questions to the family physician, (4) providing “three key questions,” and (5) getting an independent advice from the family physician. Family physicians described the TOC as a pleasant moment to talk, to check the patient’s understanding of the diagnosis and treatment, and to reflect on priorities concerning quality of life.

“Definitely, a moment of reflection and time to think about what a patient wants in life, including the related quality of life.” (Family physician)

Specialists indicated that a TOC created a moment of reflection to consider consequences and added value of therapy in the context of the patient’s personal circumstances.

### SDM step 4—Informed treatment decision

Patients responded that the TOC can influence treatment choice and can take away doubts or insecurities about treatment choice. This could entail an unchanged decision, a choice for less or no treatment, or a choice for more treatment.

“At first I didn’t want to do anything, but after the consultation (TOC) with my family physician I decided to accept chemotherapy. The birth of my grandchild also had to do with this.” (Patient)

Specialists stated that the TOC facilitated a well-considered treatment decision.

No statements addressing a negative effect of a TOC on “awareness of choice,” “deliberation,” or “informed treatment decision” were made.

### Opportunities and barriers

Patients and family physicians indicated that the family physician was better informed as a result of the TOC. Family physicians experienced more appreciation and information from the hospital and more involvement in the guidance of the patient. Family physicians mentioned that the format of the TOC and structural implementation of TOC facilitated family physicians in providing support.

A potential barrier for success is unclearness about the goal of the TOC, scheduling a TOC after treatment decision, and insufficient information exchange between specialist and family physician.

## Discussion

The first experiences with a TOC in primary care before cancer treatment decision suggest that it may help to keep the GP “in the loop” after a cancer diagnosis and that it may contribute to the SDM process, according to patients, family physicians, and specialists.

These positive experiences are in line with the results of a survey by the Dutch Federation of Cancer Patient Organizations (NFK), which shows that 66% of cancer patients indicated to want family physician support for cancer treatment decisions [9]. A recent Cochrane review summarizes the benefits of well-informed decision-making as “patients feel more knowledgeable, better informed, and clearer about their values” [10]. The observations in our evaluation confirm this.

This pragmatic assessment of a small pilot implementation does have limitations, e.g. the lack of a control arm and relatively small numbers. The results should therefore be considered explorative. Strengths of this study are the pragmatic design with implementation in a daily care setting directly reflecting impact on clinical practice and the combination of quantitative and qualitative data, which increases the understanding of the experienced added value. A strength of our pragmatic intervention is its simplicity and broad applicability. Therefore, while this study is focused on a TOC in patients with advanced disease at the initiation of therapy, there may be other decision moments throughout the cancer continuum (such as in times of diagnostic interventions) and in patients with different stages of the disease that could also benefit from a TOC. This deserves further exploration.

## Conclusion

The first experiences with offering a TOC in primary care before cancer treatment decision suggest that a TOC may help to keep the family physician in the loop after a cancer diagnosis. It may also stimulate the SDM process, thereby enabling more individualized cancer treatment decisions, according to both patients and physicians.

## Data Availability

The datasets generated during and/or analysed during the current study are available from the corresponding author on reasonable request.
